# Kentucky State Medical Society

**Published:** 1842-01

**Authors:** 


STATE MEDICAL SOCIETY OF KENUTCKY.

We beg leave to remind our Kentucky readers of the meeting of this society on the 12th inst at Frankfort. Being the fiist, it should be well attended and enriched with valuable contributions. If such should not be forthcoming, the meeting will be to little purpose.

